# Inhibition of nicotinamide phosphoribosyltransferase (NAMPT) with OT-82 induces DNA damage, cell death, and suppression of tumor growth in preclinical models of Ewing sarcoma

**DOI:** 10.1038/s41389-020-00264-0

**Published:** 2020-09-10

**Authors:** Anna E. Gibson, Choh Yeung, Sameer H. Issaq, Victor J. Collins, Michael Gouzoulis, Yiping Zhang, Jiuping Ji, Arnulfo Mendoza, Christine M. Heske

**Affiliations:** 1 Pediatric Oncology Branch, National Cancer Institute, National Institutes of Health, Bethesda, MD USA; 2 Urologic Oncology Branch, National Cancer Institute, National Institutes of Health, Bethesda, MD USA; 3 National Clinical Target Validation Laboratory, Division of Cancer Treatment and Diagnosis, National Cancer Institute, National Institutes of Health, Bethesda, MD USA

**Keywords:** Bone cancer, Cancer metabolism, Paediatric cancer, Sarcoma

## Abstract

NAMPT mediates the rate-limiting step of the NAD salvage pathway, which maintains cellular bioenergetics and provides a necessary substrate for functions essential to rapidly proliferating cancer cells. In this study, we evaluated the efficacy and mechanisms of action of OT-82, a novel, high-potency NAMPT inhibitor with a favorable toxicity profile, in preclinical models of Ewing sarcoma (EWS), an aggressive pediatric malignancy with previously reported selective sensitivity to NAMPT inhibition. We show that OT-82 decreased NAD concentration and impaired proliferation of EWS cells in a dose-dependent manner, with IC_50_ values in the single-digit nanomolar range. Notably, genetic depletion of NAMPT phenocopied pharmacological inhibition. On-target activity of OT-82 was confirmed with the addition of NMN, the product of NAMPT, which rescued NAD concentration and EWS cellular viability. Mechanistically, OT-82 treatment resulted in impaired DNA damage repair through loss of PARP activity, G2 cell-cycle arrest, and apoptosis in EWS cells. Additional consequences of OT-82 treatment included reduction of glycolytic and mitochondrial activity. In vivo, OT-82 impaired tumor growth and prolonged survival in mice bearing EWS xenografts. Importantly, antitumor effect correlated with pharmacodynamic markers of target engagement. Furthermore, combining low-dose OT-82 with low doses of agents augmenting DNA damage demonstrated enhanced antitumor activity in vitro and in vivo. Thus, OT-82 treatment represents a potential novel targeted approach for the clinical treatment of EWS.

## Introduction

Rapidly proliferating cancer cells have altered metabolic needs, including an increased rate of nicotinamide adenine dinucleotide (NAD) cycling relative to normal cells^1–3^. NAD is an essential substrate for maintaining cellular bioenergetics and supporting NAD-dependent proteins integral to DNA repair, genomic integrity, and regulation of transcription, signaling, and oxidative stress^3–5^. In several cancer types, sustained depletion of NAD has been shown to trigger apoptosis and autophagy, indicating cellular dependence on maintenance of adequate levels^6–8^.

Cellular NAD can be produced through several redundant synthesis pathways, some of which include enzymes that are over-expressed or silenced in certain cancers^3,9–16^. The salvage pathway represents one such pathway of key importance in cancer, functioning to recycle nicotinamide (NAM), the product of NAD^+^-consuming enzymes, back into NAD^+^^17^. In the salvage pathway, nicotinamide phosphoribosyltransferase (NAMPT) acts as the rate-limiting enzyme and produces nicotinamide mononucleotide (NMN), an NAD precursor^3,15–17^. In certain cancers, NAMPT expression has been shown to promote carcinogenesis and is associated with worse prognosis^3,9,16^. Preclinically, pharmacological inhibitors of NAMPT have been shown to deplete NAD, resulting in loss of cell viability in a variety of cancer types^6–8,10,18–21^. Because the cellular functions of NAD are broad, NAMPT inhibitors (NAMPTis) may have multiple anticancer effects including inhibition of energy metabolism, susceptibility to oxidative stress, and impairment of DNA damage repair^2,9,21–23^. NAMPT is currently the only NAD^+^ production enzyme that has been targeted in the clinic^2,5,24^.

First-generation NAMPTis were tested in several early phase clinical trials in unselected adult patients with advanced cancers, yielding a disease control rate of about 25% but few objective responses^25–30^. Bone marrow suppression, especially thrombocytopenia, was dose-limiting in these trials, as were gastrointestinal side-effects^25–30^. In large animal studies, retinal and cardiac toxicities were observed, although these were not reported in human patients^31,32^. Given the paucity of objective responses and concerns about NAMPTi-associated toxicities, development of this class of agents was halted^33^. OT-82 (OncoTartis) is a novel, oral, small molecule inhibitor of NAMPT currently undergoing clinical assessment for hematological malignancies. While initially discovered using an assay for hematopoietic tissue-specific cytotoxic agents, its mechanism was revealed to be a NAMPTi. Early data suggest that OT-82 possesses a more favorable toxicity profile than earlier-generation NAMPTis, particularly with regard to retinal and cardiac toxicities that were observed in animal studies of earlier-generation molecules but were not observed with OT-82^34^.

In addition, recent evidence has emerged demonstrating that certain tumor types may be more sensitive to inhibition of NAMPT due to differential vulnerabilities in NAD-related processes^9^. Ewing sarcoma (EWS), a pediatric bone and soft tissue cancer, represents one such malignancy as recent studies have revealed the presence of intrinsic defects in DNA damage repair and metabolic reprogramming^35–39^. Furthermore, in vitro data using early-generation NAMPTis suggests that EWS cells may be more sensitive than other cancer cell types^40,41^. However, since EWS patients were never treated in any early NAMPTi clinical trials, the potential clinical efficacy of this class of agents remains untested in this population. Thus, the purpose of this study was to evaluate the activity and mechanistic effects of the latest-generation NAMPT inhibitor OT-82 in in vitro and in vivo models of EWS.

## Results

### NAMPT is a critical enzyme for EWS cell growth and survival that can be inhibited by OT-82

**Fig. 1:**
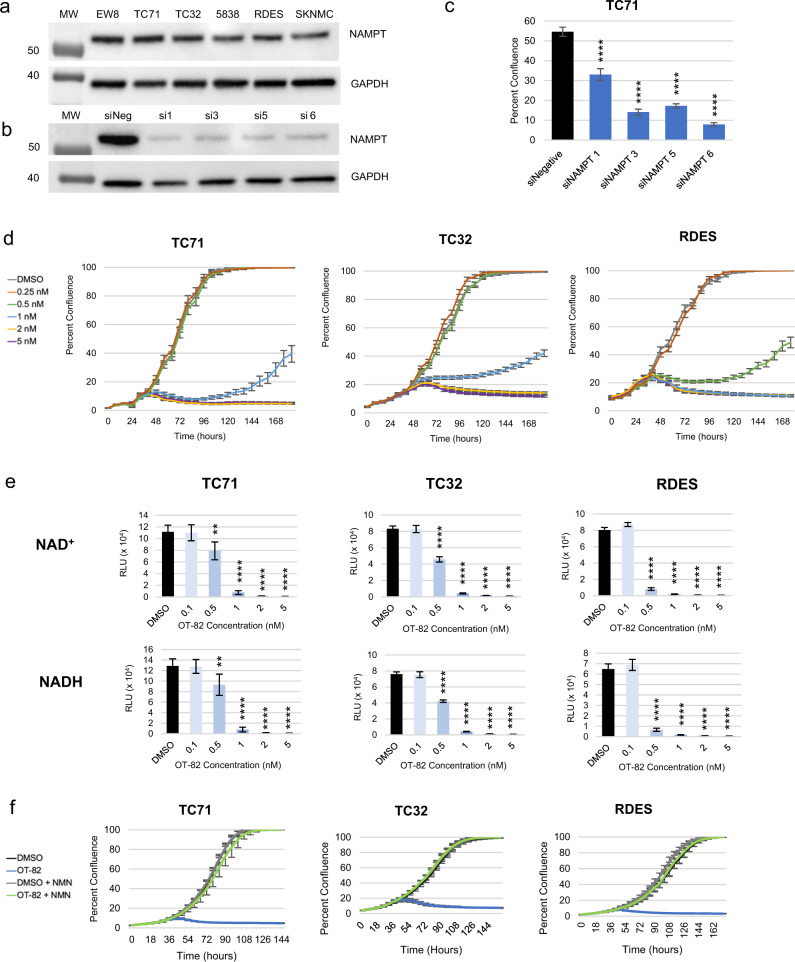
Ewing sarcoma (EWS) cells are sensitive to genetic and pharmacological inhibition of NAMPT with OT-82, an efficacious, on-target NAMPT inhibitor. **a** Immunoblot analysis of NAMPT expression in EWS cell lines. **b** Immunoblot analysis of NAMPT expression in TC71 72 h after NAMPT siRNA knockdown using multiple sequences. **c** Cellular viability, represented by percent confluence, 6 days after NAMPT siRNA transfection using multiple sequences in TC71. Quadruple asterisks (****) denote *p* < 0.0001 for one-way ANOVA with Dunnett’s multiple comparisons test, as compared to siNegative control. **d** IncuCyte live-cell analysis of EWS cell lines (TC71, TC32, and RDES) treated with 0.2–5 nM OT-82. **e** NAD^+^ and NADH concentrations in EWS cells treated with DMSO or 0.1–5 nM OT-82 for 72 h. Double asterisks (**) denote *p* < 0.01, quadruple asterisks (****) denote *p* < 0.0001 for one-way ANOVA with Dunnett’s multiple comparisons test. **f** IncuCyte live-cell analysis of EWS cell lines treated with DMSO or 10 nM OT-82 in the presence or absence of 1 mM NMN.

To first determine the importance of NAMPT in EWS, we investigated a panel of EWS cell lines for NAMPT expression and dependency on NAMPT. All six cell lines representing a range of molecular features (Supplementary Table 1) expressed NAMPT at nearly equivalent levels (Fig. 1a). Genetic depletion of NAMPT using multiple distinct siRNA sequences in TC71 EWS cells resulted in loss of NAMPT expression and significant inhibition of cell growth, measured by IncuCyte live-cell analysis (Fig. 1b, c and Supplementary Fig. S1a). Additional molecularly-heterogenous EWS cell lines (TC32 and RDES) showed similar results using the most effective sequence, compared to positive and negative controls (Supplementary Fig. S1b, c, and d).

We next used the novel, clinically relevant NAMPTi OT-82 to determine the effect of pharmacological inhibition of NAMPT on EWS cell lines. The use of OT-82 recapitulated the genetic findings, with dose-dependent growth inhibition and morphologic changes observed via IncuCyte live-cell analysis. OT-82 was highly potent, with complete growth inhibition observed at doses between 0.5 and 2 nM in most EWS cell lines (Fig. 1d and Supplementary Fig. S1e, f). Notably, the potency of OT-82 was equal to or greater than that of the earlier-generation NAMPTis FK-866 and GNE-618, which typically required at least 5 nM to completely suppress cellular proliferation (Supplementary Fig. S2a).

The primary expected consequence of loss of NAMPT activity is a reduction in the cellular production of NAD, thus, we next characterized the effect of OT-82 on intracellular NAD^+^ and NADH levels. We observed dose-dependent reductions in both intracellular NAD^+^ and NADH in EWS cells upon treatment with OT-82 for 24 and 72 h, with complete loss of NAD at 1 nM by 72 h (Fig. 1e and Supplementary Fig. S2b). Importantly, the degree of NAD^+^ and NADH loss at a particular dose correlated with the effects on cellular viability. Compared to earlier-generation NAMPTis, OT-82 treatment resulted in an equal or greater degree of NAD depletion than FK-866 and GNE-618, respectively (Supplementary Fig. S2c). Addition of nicotinamide mononucleotide (NMN), the product of NAMPT, to EWS cells at the time of OT-82 treatment resulted in rescue of NAD levels and reversed the antiproliferative effects of high dose OT-82 on EWS cells, confirming its on-target activity (Fig. 1f and Supplementary Fig. S3a, b). Taken together, these findings indicate that OT-82 acts as a potent inhibitor of NAMPT in EWS cells, decreasing cellular NAD^+^ and NADH, and inhibiting proliferation.

### OT-82 decreases PARP activity and results in increased DNA damage in EWS cells

**Fig. 2:**
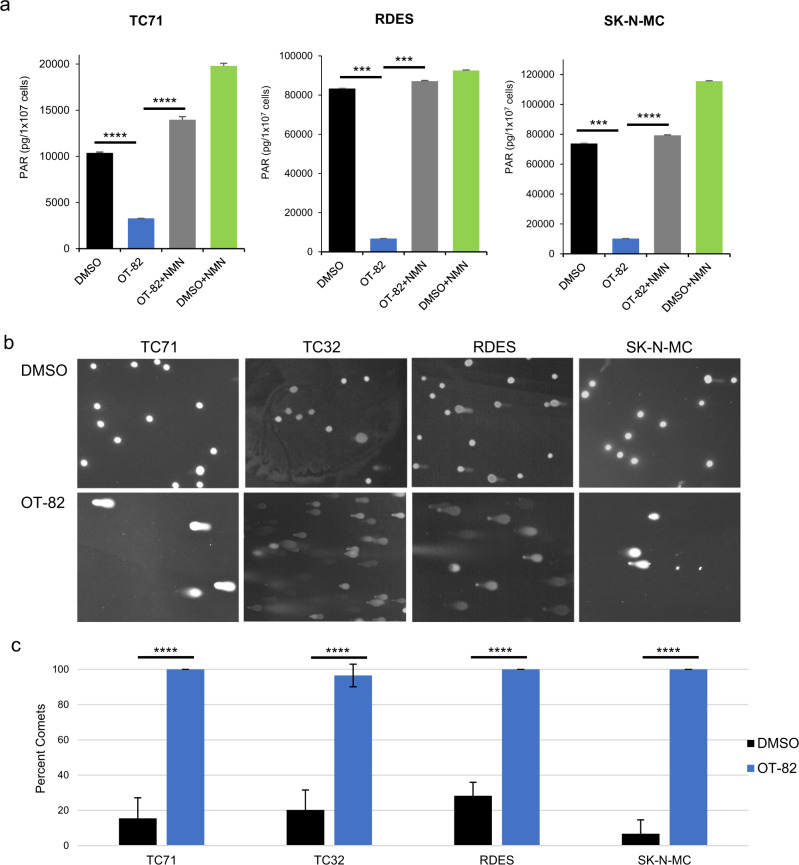
OT-82 treatment results in decreased PARP activity and increased DNA damage in EWS cells. **a** PAR levels in EWS cell lines treated with DMSO or OT-82 (5 nM) in the presence or absence of 1 mM NMN for 24 h. Triple asterisks (***) denote *p* < 0.001, quadruple asterisks (****) denote *p* < 0.0001**. b** Comet assay representative images of EWS cells treated with DMSO or OT-82 (5 nM) for 72 h. **c** Quantification of images represented in panel **b**. quadruple asterisks (****) denote *p* < 0.0001.

A key expected consequence of cellular NAD^+^ depletion is the loss of enzymatic activity of NAD^+^-consuming enzymes^9^. Poly (ADP-ribose) polymerases (PARPs) comprise one such group of enzymes, which are known to be major users of cellular NAD^+^ and are of critical importance in DNA damage repair^42,43^. To assess the impact of OT-82 on PARP-mediated DNA repair, we measured the effect of OT-82 on PARP activity and DNA damage in EWS cells. Following 24 h of treatment with OT-82, EWS cells displayed a significant decrease in PARP activity, which was rescued with the coadministration of NMN (Fig. 2a). Interestingly, addition of NMN alone increased baseline PARP activity in the EWS cell lines tested, suggesting that NAD^+^ may be a limiting factor for PARP activity in EWS cells. Notably, the dose of OT-82 required to achieve a similar level of PARP inhibition as the PARP inhibitor niraparib was 200-fold lower (Supplementary Fig. S4). Since the expected functional consequence of diminished PARP activity is an increase in DNA damage, we next evaluated treated cells for increased DNA damage. Indeed, EWS cells treated with OT-82 demonstrated a four-fold increase in DNA damage, measured by comet assay (Fig. 2b, c).

### OT-82 induces G2 arrest and apoptotic cell death in EWS cells

**Fig. 3:**
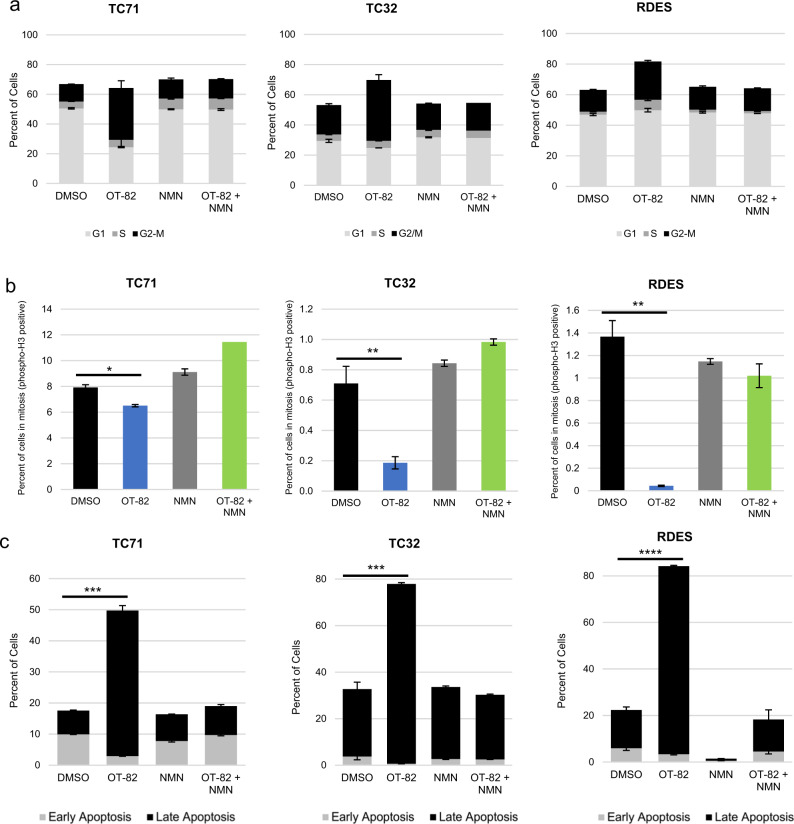
OT-82 induces G2 arrest and apoptotic cell death in EWS cells. **a** Cell-cycle analysis in EWS cell lines in the presence and absence of NMN (1 mM) as determined by flow cytometric analysis of DNA content after 72 h of treatment with OT-82 (5 nM). **b** Phosphorylated histone H3 staining after 72 h of treatment with OT-82 (5 nM) in EWS cell lines as determined by flow cytometry. Single asterisk (*) denotes *p* < 0.05, double asterisks (**) denote *p* < 0.01. **c** Percentage of EWS cells in early (Annexin V–positive, PI-negative) and late (Annexin V–positive, PI-positive) apoptosis following treatment with DMSO or OT-82 (5 nM) in the presence or absence of 1 mM NMN for 72 h, as determined by flow cytometry. Triple asterisks (***) denote *p* < 0.001, quadruple asterisks (****) denote *p* < 0.0001 for comparisons of late apoptosis values.

To determine the mechanism through which OT-82 affects cellular proliferation of EWS cells, we next characterized the effects of OT-82 on the cell cycle. DNA content analysis of EWS cells treated with OT-82 demonstrated at least a doubling in the percentage of cells in the G2/M phase compared to control treatment. Coadministration of NMN reversed this effect (Fig. 3a and Supplemental Fig. S5a). To determine whether the cells were arresting in G2 or M phase, we next examined the effect of OT-82 on phosphorylated histone H3 (PHH3), a marker of cells undergoing mitosis. We found that expression of PHH3 was significantly decreased in cells treated with OT-82, indicating that OT-82 treatment prevents cellular entry into mitosis. This effect was similarly reversed with the coadministration of NMN (Fig. 3b and Supplemental Fig. S5b). To understand the effect of OT-82 on EWS cell viability, we analyzed OT-82-treated cells by Annexin-V/propidium iodide staining. OT-82 treatment resulted in a two- to six-fold increase in the percentage of EWS cells in late apoptosis, depending on the cell line, which was reversible with coadministration of NMN (Fig. 3c and Supplemental Fig. S5c). Taken together, these findings suggest that OT-82 has both a cytostatic and cytotoxic effect on EWS cells.

### OT-82 affects additional NAD^+^-dependent processes in EWS cells

**Fig. 4:**
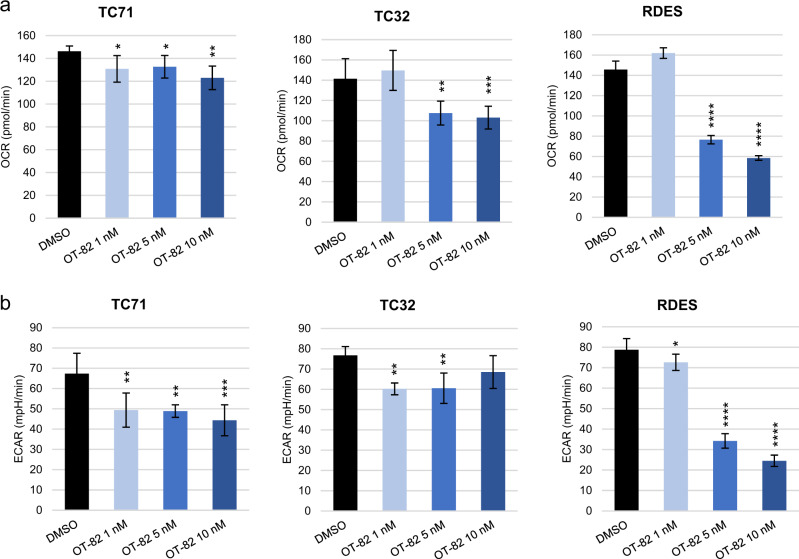
Diminished mitochondrial and glycolytic activity are additional downstream consequences of OT-82 treatment in EWS cells. **a** Oxygen consumption rate (OCR) for EWS cells after treatment with OT-82 at 1 nM, 5 nM, or 10 nM. Single asterisk (*) denotes *p* < 0.05, double asterisks (**) denote *p* < 0.01, quadruple asterisks (****) denote *p* < 0.0001, for one-way ANOVA with Dunnett’s multiple comparisons test, comparing to control. **b** Extracellular acidification rate (ECAR) for EWS cells after treatment with OT-82 at 1 nM, 5 nM, or 10 nM. Single asterisk (*) denotes *p* < 0.05, double asterisks (**) denote *p* < 0.01, triple asterisks (***) denote *p* < 0.001, quadruple asterisks (****) denote *p* < 0.0001, for one-way ANOVA with Dunnett’s multiple comparisons test, comparing to control.

Given the important role of NAD^+^ homeostasis in additional cellular functions besides DNA damage repair, such as maintenance of energy metabolism and management of oxidative stress^2,9,21,22^, we next investigated the effects of OT-82 treatment on these processes in EWS cells. We hypothesized, based on recent data showing that early-generation NAMPTis impaired bioenergetics in EWS cells^40^, that OT-82 would have a similar effect. Using extracellular flux analysis, we performed measurements of oxygen consumption rate (OCR) and extracellular acidification rate (ECAR) in EWS cells following treatment with OT-82. Significant dose-dependent decreases in both OCR and ECAR were observed to varying degrees in each of the EWS cell lines tested, suggesting that OT-82 reduces both oxidative phosphorylation (Fig. 4a) and glycolysis (Fig. 4b) in EWS cells. Given the established antiproliferative effect that results from inhibiting glycolysis and oxidative phosphorylation in EWS cells^37,44^, the bioenergetic effects of OT-82 likely contribute, at least in part, to its negative effect on proliferation.

Induction of reactive oxygen species (ROS) is another commonly reported downstream effect of NAMPT inhibition^45–50^. While treatment with OT-82 resulted in at least a six-fold increase in ROS in EWS cells, addition of the antioxidant N-acetylcysteine (NAC) did not rescue cellular viability, despite reducing ROS to baseline levels, and suggests that ROS induction is not driving the antiproliferative effects of OT-82 (Supplementary Fig. S6a, b and c). This finding is consistent with our prior work using other NAMPTis in EWS cell lines^41^.

### OT-82 impairs tumor growth and prolongs survival in EWS xenograft models

**Fig. 5:**
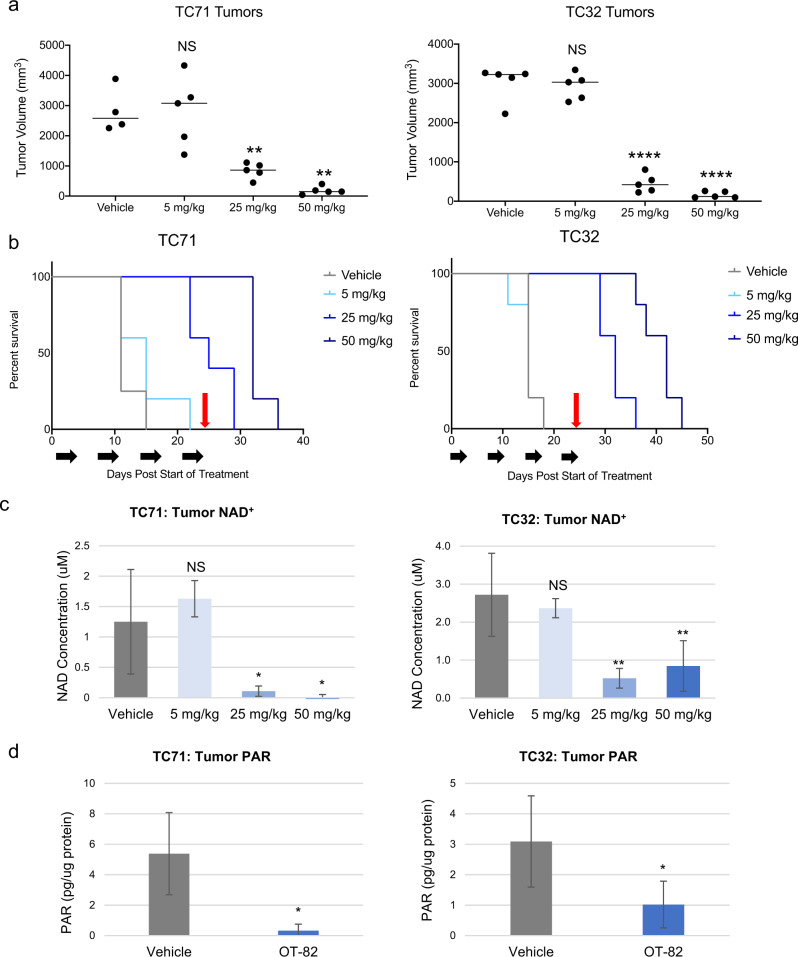
OT-82 impairs tumor growth and prolongs survival in EWS orthotopic xenograft models. **a** EWS xenograft tumor volumes on days 15 (TC71) and 18 (TC32) after treatment with vehicle or OT-82 at 5, 25, or 50 mg/kg. Animals (*n* = 5/group) were dosed with OT-82 on days 0–2, 7–9, 14–16, and 21–24. Double asterisks (**) denote *p* < 0.01, quadruple asterisks (****) denote *p* < 0.0001**. b** Corresponding Kaplan–Meier curves representing survival to endpoint (17 mm in longest tumor diameter) for mice bearing EWS xenograft tumors and treated with vehicle or 5, 25, or 50 mg/kg OT-82 as described in **a**. Black arrows represent treatment schedule; red arrows represent final day of treatment. In TC71 xenografts, *p* = 0.0093 for 25 mg/kg compared to control and *p* = 0.0050 for 50 mg/kg compared to control; in TC32 xenografts, *p* < 0.0001 for both 25 mg/kg and 50 mg/kg compared to control, using Mantel–Cox analysis. **c** Total NAD concentration in EWS xenograft tumors (*n* = 3 mice/condition) treated with vehicle or OT-82 at 5, 25 or 50 mg/kg for 3 days. Tumors were harvested 2 h after third dose. Double asterisks (**) denote *p* < 0.01, single asterisk (*) denotes *p* < 0.05. **d** PAR activity in EWS xenograft tumors (*n* = 3 mice/condition) treated with vehicle or 50 mg/kg OT-82 for 3 days. Tumors were harvested 2 h after third dose. Single asterisk (*) denotes *p* < 0.05.

Given the profound sensitivity of EWS cells to OT-82 in vitro, we next sought to determine whether OT-82 would retain efficacy in vivo. This is of particular importance for the preclinical study of metabolic agents and for agents targeting solid tumors, given the complex metabolic and microenvironmental factors that cannot be duplicated in vitro^51^. Using orally administered OT-82 given on the same 3-day-on, 4-day-off schedule being used in the phase 1 first-in-human study (NCT03921879), we tested a range of doses (5, 25, and 50 mg/kg) in two orthotopic xenograft models of EWS. On this schedule, OT-82 treatment resulted in significantly reduced tumor volumes for mice treated at the two higher doses in both models (Fig. 5a and Supplementary Fig. S7a). Correspondingly, survival was significantly improved in a dose-dependent manner in both models at the two higher doses (Fig. 5b). Mice maintained optimal general appearance and body weight at all doses throughout the study, indicating no overt toxicity associated with OT-82 administration (Supplementary Fig. S7b).

Pharmacodynamic assessment of total NAD levels in tumors harvested from animals after 3 days of treatment with vehicle or one of three dose levels of OT-82 demonstrated at least a four-fold decrease in total intra-tumoral NAD in both models for the mice treated at 25 or 50 mg/kg (Fig. 5c). Importantly, the groups that experienced a significant reduction in total NAD were those that experienced antitumor activity and a survival advantage, confirming the importance of NAD for tumor growth. Additional pharmacodynamic assessment using PARP activity in tumors from animals treated at 50 mg/kg demonstrated that the activity of this NAD^+^-consuming enzyme was significantly decreased by OT-82 in vivo (Fig. 5d). This indicates that PARP activity represents an additional pharmacodynamic marker of NAMPT inhibitor activity, and suggests that this type of assay, such as those currently used in PARP inhibitor clinical trials, could be of use to demonstrate target engagement in trials testing OT-82.

### In vivo activity of OT-82 is enhanced by addition of agents augmenting DNA damage

**Fig. 6:**
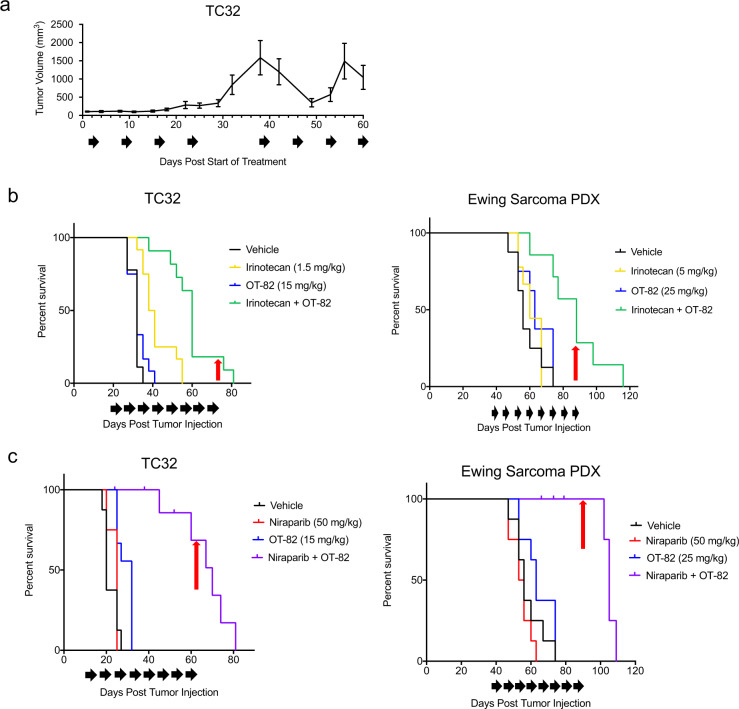
Combination treatments with agents that augment DNA damage improve single-agent efficacy of low-dose OT-82 in EWS orthotopic xenograft models. **a** Tumor growth curve for TC32-bearing xenografts (*n* = 3) treated with 50 mg/kg OT-82 over an initial 4 week treatment period (mice treated days 1–3, 8–10, 15–17, and 22–24), a 2-week treatment interruption, and an additional 4 week treatment period (mice treated days 38–40, 45–47, 52–54, and 59–60). Black arrows represent treatment schedule. **b** Kaplan–Meier curves representing survival to endpoint (17 mm in longest tumor diameter) for mice (*n* = 12/group) bearing EWS xenograft tumors (TC32 and PDX) and treated with vehicle or the indicated doses of OT-82, irinotecan (daily on 2 of every 7 days for TC32; 5 of every 7 days for PDX) or the combination. Black arrows represent treatment schedule; red arrows represent final day of treatment. In TC32 xenografts, *p* = 0.0004 for irinotecan compared to combination, *p* < 0.0001 for OT-82 compared to combination; in the PDX, *p* = 0.0018 for irinotecan compared to combination, *p* = 0.0065 for OT-82 compared to combination using Mantel–Cox analysis. c Kaplan–Meier curves representing survival to endpoint (17 mm in longest tumor diameter) for mice (*n* = 12/group) bearing EWS xenograft tumors (TC32 and PDX) and treated with vehicle, or the indicated doses of OT-82, niraparib (daily on 5 of every 7 days) or the combination. Black arrows represent treatment schedule; red arrows represent final day of treatment. In TC32 xenografts, *p* = 0.0001 for niraparib compared to combination, *p* < 0.0001 for OT-82 compared to combination; in the PDX, *p* < 0.0001 for niraparib compared to combination, *p* = 0.0003 for OT-82 compared to combination using Mantel–Cox analysis.

Single-agent treatment with OT-82 demonstrated robust antitumor activity, however cessation of treatment resulted in tumor regrowth, supporting, at least in part, a cytostatic mechanism of action in vivo. Retreatment of large, recurrent tumors following a 2-week treatment break resulted in tumor regressions; however, this response was less durable (Fig. 6a). Given this, and the fact that no single-agent treatment has been shown to produce durable responses in patients with EWS, we next sought to test the effect of using OT-82 in rational combinations. Chemotherapeutic agents currently comprise the standard upfront and relapse regimens for EWS^52^. In light of the mechanistic effect of OT-82 on DNA repair, we hypothesized that OT-82 could enhance the antiproliferative effects of chemotherapy. Thus, we first tested in vitro combinations of OT-82 with a selection of standard-of-care chemotherapeutic agents used against EWS and representing distinct mechanisms of action in EWS cell lines. The combination of low-dose OT-82 with the tubulin inhibitor vincristine, the alkylating agent doxorubicin, or the topoisomerase-2 inhibitor etoposide did not demonstrate appreciable additive activity at the doses tested (Supplementary Fig. S8). However, the combination of OT-82 with the topoisomerase-1 inhibitor SN-38 resulted in enhanced antiproliferative effects using minimally active doses of each single agent in most EWS cell lines (Supplementary Figs. S8 and S9).

Given the activity of OT-82 plus SN-38 observed in vitro and previous studies supporting the enhanced antitumor effect of topoisomerase-1 inhibitors in combination with inhibition of PARP^53,54^, we selected irinotecan, a prodrug of SN-38 and a standard agent for EWS in the relapse setting, for further evaluation in combination with OT-82 in vivo. We used clinically relevant doses of irinotecan that were at or below those equivalent to human clinical doses^36^ and tested two different schedules, the protracted 5-day per week schedule typically used in pediatrics^55^ and a less frequent, 2-day per week schedule that delivered a lower cumulative weekly dose. In both models, the combination was more efficacious than either single-agent alone and slowed tumor growth. For the orthotopic cell line xenograft model, OT-82 plus twice weekly irinotecan extended median survival from 32 (OT-82) to 60 days (combination); for the patient-derived xenograft (PDX) model, OT-82 plus daily protracted irinotecan increased median survival from 63 (OT-82) to 88 days (combination) (Fig. 6b and Supplementary Fig. S10a). Notably, mice maintained adequate body weight, activity, and appearance throughout the treatments (Supplementary Fig. S10b).

Finally, given previous work identifying synergistic activity between early-generation NAMPTis and PARP inhibitors^41^, we sought to determine whether OT-82 antiproliferative activity could similarly be enhanced when combined with clinically relevant doses of niraparib^56,57^, a clinical PARP inhibitor. In vitro, subtherapeutic doses of niraparib and OT-82 resulted in complete inhibition of cellular proliferation in EWS cell lines (Supplementary Fig. S11a). Likewise, in vivo testing of this combination in TC32 and a PDX resulted in tumor growth inhibition and a significant extension of survival from 32 (OT-82) to 69 days (combination) for the TC32 xenograft and from 63 (OT-82) to 105 days (combination) for the PDX (Fig. 6c and Supplementary Fig. S11b). Although minimal body weight loss was observed with this combination (Supplementary Fig. S11c), several unexpected deaths occurred in the combination treated groups, starting after the fourth week of treatment, suggesting that prolonged combination of these agents may increase toxicity.

## Discussion

In this study we show that loss of NAMPT activity through genetic or pharmacological inhibition results in impaired EWS cell proliferation and survival, demonstrating the dependency of EWS on NAMPT. We provide the first evaluation in solid tumor models of the activity of OT-82, a recently described, novel NAMPTi that was selected for clinical development due to specific cytotoxicity against hematologic malignancies^34^. Remarkably, we observed equal or lower IC_50_ values for OT-82 in our panel of EWS cell lines than those reported for the preclinical models of hematologic malignancies, suggesting that EWS may represent a selectively sensitive candidate solid tumor^34,58^. The enhanced sensitivity of EWS to NAMPT inhibition may be, in part, related to the known homologous repair deficiency that is a feature of EWS^35^, as DNA repair deficiencies have been linked to NAMPTi sensitivity in other solid tumor types^23^ and may increase dependence on the NAD-dependent PARPs^59,60^. Other factors specific to EWS may also contribute to NAMPTi sensitivity, including the presence of the oncogenic fusion protein EWS-FLI1^40^, which has been shown to drive reprogramming of NAD-dependent metabolic enzymes in EWS, such as PHGDH and LDHA^37–39,44^, and reliance on other NAD-dependent proteins such as SIRT1^61,62^.

Our mechanistic studies of OT-82 in EWS indicate that it functions by depleting cellular NAD, which in turn results in both G2 arrest and apoptosis. This is partially consistent with its effect in leukemia models, where OT-82 was observed to induce apoptosis, but not cell-cycle arrest^34,58^. Interestingly, use of the earlier-generation NAMPTi FK-866 has been associated with G2/M arrest in models of other solid tumors^63^, suggesting that the specific mechanistic effects of NAMPT inhibition may be cell-type specific. The effect of OT-82 on ROS induction in our models represents an additional example of the differential effects NAMPTis may have on different cell types. Induction of ROS is a known mechanism of NAMPTi-induced killing in a range of cancer cell types^45–50^. However, as we demonstrated in this study, induction of ROS was not responsible for OT-82-induced cell death in EWS, suggesting that ROS production is a byproduct of OT-82-induced cell death. This is in contrast to the effect of OT-82-induced ROS reported in PDX models of pediatric acute lymphoblastic leukemia^58^ but is concordant with prior results using the NAMPTi GNE-618 in EWS^41^, and suggests that the differential effects are due to the affected cell type, as opposed to the particular NAMPTi used.

In vivo, OT-82 treatment resulted in substantial inhibition of EWS xenograft tumor growth and prolonged survival at doses demonstrating pharmacodynamic evidence of on-target activity in tumors, using NAD concentration and PARP activity. Notably, PARP activity assays have been successfully used in the clinic^64^ and therefore represent a highly quantitative assay for on-target NAMPT inhibition in EWS that could potentially be incorporated in future clinical trials. Intermittently treated tumors retained sensitivity to OT-82, however, sustained durability of response was not observed using an intermittent schedule. This appears to be consistent with the data describing single-agent OT-82 use in most in vivo leukemia models, although the results of prolonged intermittent dosing were not reported in those studies^34,58^. These findings suggest that a subpopulation of cells may be resistant, although the underlying mechanisms responsible for this are not yet clear. Tumor heterogeneity, incomplete or inconsistent drug delivery to certain areas of the tumor, or apoptotic resistance, all of which have been described as mechanisms of resistance for other anticancer agents^65–67^ may contribute to our understanding of these results and are important future areas of investigation.

The addition of DNA damage-augmenting agents (irinotecan and niraparib), which are clinically relevant agents for EWS, enhanced both disease control and animal survival in our models, suggesting that clinically, OT-82 would optimally be used as part of a combination regimen. While single-agent OT-82 and OT-82 plus niraparib both resulted in tumor regression in the in vivo models tested, the addition of low-dose irinotecan to OT-82 slowed tumor growth but surprisingly did not result in tumor regressions. This may suggest that a higher dose of irinotecan is required for such an effect and/or that the drug dosing schedule and/or sequencing of agents may be important. In addition, while our experiments used clinically relevant doses of irinotecan and niraparib that were guided by existing human pharmacokinetic (PK) data, human PK data on OT-82 are not yet available. These forthcoming data represent an additional important factor in assessing the translational potential of OT-82. Further preclinical investigation should focus on establishing and optimizing effective combination regimens using clinically achievable drug concentrations that result in durable responses in preclinical models. Consideration of the human toxicity profile of OT-82 and whether it overlaps with the toxicity profiles of potential combination partners will also be of utmost importance.

In conclusion, our results demonstrate that dependence on NAMPT represents a therapeutic vulnerability in EWS, and thus, that OT-82, with its improved toxicity profile, may be a promising new agent for the treatment of EWS. These findings provide a rationale for early phase testing of this drug in this patient population.

## Materials and methods

### Cell lines

EWS cell lines (TC71, TC32, RDES, EW8, SK-N-MC, CHLA-258, and 5838) have been previously described^44^. Mycoplasma testing was most recently performed in February 2020 and confirmed negative. Cells were maintained in RPMI growth medium (Life Technologies, Grand Island, NY) with 10% FBS, heat-inactivated (MilliporeSigma, St. Louis, MO), 100 U/mL penicillin and 100 μg/mL streptomycin (Life Technologies), and 2 mmol/L l-glutamine (Life Technologies) at 37 °C in an atmosphere of 5% CO_2_.

### Compounds

OT-82 was provided by OncoTartis (Buffalo, NY). FK-866, GNE-618, and niraparib were obtained from Dr. Craig Thomas (National Center for Advancing Translational Science, Rockville, MD). NMN and NA were obtained from MilliporeSigma. SN-38 was obtained from SelleckChem (Houston, TX). Irinotecan was obtained from the National Institutes of Health (NIH) Veterinary Pharmacy (Bethesda, MD). Details of drug preparation can be found in Supplemental Methods.

### Immunoblotting

Details of cell harvest and lysate preparation can be found in Supplemental Methods. Blots were incubated with antibody against NAMPT (ProteinTech, Rosemont, IL). GAPDH (Santa Cruz Biotechnology, Dallas, TX) was used as the loading control. Bands were visualized on a BioRad Image Lab camera using West Femto and Pico ECL detection reagent (ThermoFisher Scientific, Grand Island, NY).

### siRNA

Lipofectamine RNAiMAX (ThermoFisher) was used according to the manufacturer’s instructions to deliver 25 pM siNAMPT (Qiagen: Hs_PBEF1_1,3,5,6) to 250,000 cells/well in six-well plates, 24 h after seeding. Protein was harvested 72 h post-transfection in 1X RIPA buffer (Santa Cruz Biotechnology) for western blot. For proliferation assays, cells were plated at 10 000 cells/well in six-well plates and siRNA was delivered as described above when cells had reached 20% confluency for TC71/TC32, and 30% confluency for RDES. Proliferation assays were repeated at least three times. Between 4- and 6-days post-transfection, cells were stained with NeatStain (AstralDiagnostics, West Deptford, NJ) and images were acquired.

### Cell proliferation assays

The IncuCyte live-cell analysis system (Essen BioScience, Ann Arbor, MI) was used to assess proliferation of EWS cell lines in real time. 2000 cells/well were plated in 96-well plates, allowed to adhere overnight, and treated the following day, unless otherwise indicated. Each experiment was performed at least two times with three biological replicates.

### NAD/NADH analysis

Cells were plated at either 5000 or 2500 cells/well in 96-well plates, allowed to adhere overnight and treated with OT-82± NMN for 24 or 72 h, respectively. NAD^+^/NADH concentrations were determined using the NAD/NADH-Glo Assay (Promega, Madison, WI) per manufacturer’s instructions. Each experiment included at least three biological replicates.

### PAR immunoassay

Details of cell and tissue treatments, harvest and preparation can be found in Supplemental Methods. The validated chemiluminescent immunoassay for PAR using commercially-available anti-PAR mouse monoclonal antibody (clone 10 H; Trevigen, Gaithersburg, MD) has been previously described^41,68,69^. Cell-based experiments included three technical replicates per condition. Tumor-based experiments included five biological replicates (5 mice) per condition.

### Comet assay

Cells were plated at 10^6^/10-cm plate overnight before treatment with DMSO or OT-82 for 72 h. Adherent cells were detached using 0.05% trypsin (ThermoFisher), counted and resuspended at 10^5^ cells/ml in cold PBS (ThermoFisher). Cells were analyzed with the CometAssay Silver Kit (Trevigen) per manufacturer’s instructions using the Alkaline CometAssay protocol. Samples were viewed using a Nikon Eclipse TE300 microscope. Each experiment was performed at least two times. For each condition, images of at least six different frames were viewed to manually quantify percentage of comet tails.

### Cell-cycle analysis

Details of cell harvest and preparation can be found in Supplemental Methods. Samples were run on an LSRFortessa flow cytometer (BD Biosciences, San Jose, CA) and analyzed with FlowJo software (Vancouver, Canada). Each experiment was performed at least two times with three technical replicates.

### Phospho-histone H3 (Ser10) staining

Details of cell harvest and preparation can be found in Supplemental Methods. Samples were run on an LSRFortessa flow cytometer (BD Biosciences). Each experiment was performed at least two times with three technical replicates.

### Annexin V assay

Cells were plated at 10^6^/10-cm plate overnight before treatment. Cells were harvested at 72 h and processed according to the annexin V-FITC apoptosis detection kit (MilliporeSigma) before being run on the LSRFortessa flow cytometer (BD Biosciences). Each experiment was performed at least two times with three technical replicates.

### Extracellular flux analysis

Analyses of cellular bioenergetics were performed using the Seahorse XF^e^96 Extracellular Flux Analyzer (Agilent, Santa Clara, CA). Twenty-five thousands cells/well were plated in XF 96-well plates (Agilent) and allowed to adhere at 37 °C, 5% CO_2_, for 7 h before treatment with OT-82 or DMSO for 16 h. Measurements were performed according to manufacturer’s instructions, as previously described^44,70,71^. Each experiment was performed at least three times with three technical replicates.

### ROS analysis

Two thousand five hundred cells/well were plated in white 96-well plates (PerkinElmer, Waltham, MA) and treated the following day with DMSO, OT-82, NMN, or the combination. N-acetylcysteine (Sigma) was pH-adjusted to 7.8 in water and added to cells. Cells were processed according to the ROS-Glo assay protocol (Promega). Luciferase activity was measured with a Molecular Device SpectraMax M3 plate reader. Each experiment was performed at least two times with three technical replicates.

### Statistical analyses

Statistical significance between two groups was determined using a Student *t-*test. For comparisons of three or more groups, a one-way ANOVA with Dunnett’s multiple comparisons test was used. *P* < 0.05 was considered significant.

### In vivo studies

#### Animal studies

Animal studies were performed in accordance with the guidelines of the NIH Animal Care and Use Committee. Four- to six-week-old female Fox Chase SCID beige mice (CB17.B6-Prkdcscid Lystbg/Crl) from Charles River Laboratories (Wilmington, MA) or NOG-F Homozygous/Homozygous NOD.Cg-*Prkdc*^*scid*^ *Il2rg*^*tm1Wjl*^/SzJ mice from the Jackson Laboratory (Bar Harbor, ME) were used for cell line xenografts or PDX experiments, respectively. The transplantable xenograft of primary human EWS (XEN-EWS-021) was previously described^72^.

Details of cell preparation and injection and treatments can be found in Supplemental Methods. Mice were randomized after tumors were palpable, without blinding. Single-agent experiments had five mice/group and combination experiments had 12 mice/group. Mice were maintained in a pathogen-free environment and monitored by observation and weekly weights. Tumors were measured twice/week with calipers. Tumor volume was calculated by the formula: V(mm^3^) = (D × d^2^)/6 × 3.14, where D and d represent the longest and shortest tumor axis, respectively.

#### Pharmacodynamic analysis

Interim tumors were harvested two hours after the third dose of three consecutive days of OT-82 treatment (at least three mice/condition). Tissue was immediately flash-frozen in liquid nitrogen and processed using a Dounce Homogenizer. NAD analysis was performed using the NAD/NADH Assay Kit (Abcam) per manufacturer’s instructions. PARP activity was analyzed as described in the in vitro section.

### Statistical analyses

Tumor volumes were compared between groups using a nonparametric *t*-test at indicated time points. Mantel–Cox analysis was performed to compare survival of mice. Statistical significance was defined as *p* < 0.05.

## Supplementary information

Supplemental Table, Figures, and Legends

Supplemental Methods
