# Supplementary material for: Inhibition of nicotinamide phosphoribosyltransferase (NAMPT) with OT-82 induces DNA damage, cell death, and suppression of tumor growth in preclinical models of Ewing sarcoma
Source: Oncogenesis. 2020 Sep 10;9(9):80. doi: 10.1038/s41389-020-00264-0 (PMC7481307; doi:10.1038/s41389-020-00264-0)
Supplement: Supplementary file 1 — Supplemental Table, Figures, and Legends [file 41389_2020_264_MOESM1_ESM.pdf]

Inhibition of nicotinamide phosphoribosyltransferase (NAMPT) with OT-82 induces DNA damage, cell death, and suppression of tumor growth in preclinical models of Ewing sarcoma

Anna E. Gibson, Choh Yeung, Sameer H. Issaq, Victor J. Collins, Michael Gouzoulis, Yiping Zhang, Jiuping Ji, Arnulfo Mendoza, Christine M. Heske

Supplemental Material

Supplementary Table 1. Molecular characteristics of Ewing sarcoma cell lines. Fusion type, TP53 mutations, STAG2 mutations and CDKN2A mutations detected in each cell line from published data. Empty boxes indicate no mutations were detected and/or protein was detected.

Cell Line	Fusion Type	TP53 Status	STAG2 Status	CDKN2A Status
TC71[1]	EWS-FLI1 Type I	R213X & G245C – No protein		Homozygous deletion
TC32[1]	EWS-FLI1 Type I		Y636fs – No protein	Homozygous deletion
RDES[1]	EWS-FLI1 Type II	R273C		
SK-N-MC[1]	EWS-FLI1 Type I	Expression loss – No protein		
EW8[1]	EWS-FLI1 Type I	Y220C – No protein	N475fs – No protein	
5838[2]	EWS-ERG	Unknown	Unknown	
CHLA-258[1]	EWS-FLI1 Type III	No protein		Homozygous deletion

[1] Brohl et al, PLoS Genet, 2014.
[2] Dunn et al, Cancer Genet Cytogenet, 1994.

Fig. S1

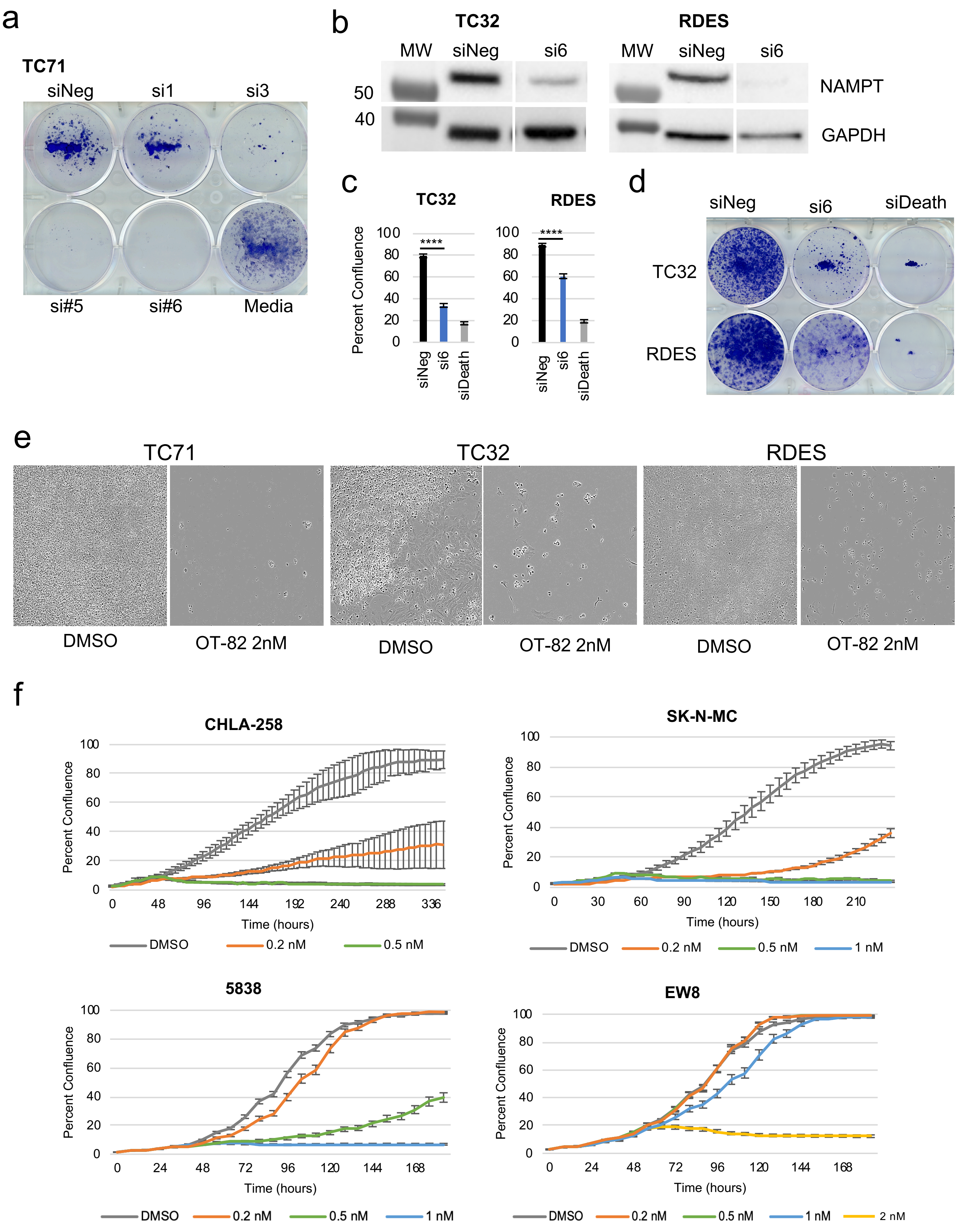

a
TC71
siNeg
si1
si3
si#5
si#6
Media
b
TC32
RDES
MW
siNeg
si6
50
40
NAMPT
GAPDH
c
TC32
RDES
Percent Confluence
100
80
60
40
20
0
****
siNeg
si6
siDeath
d
siNeg
si6
siDeath
TC32
RDES
e
TC71
TC32
RDES
DMSO
OT-82 2nM
f
CHLA-258
SK-N-MC
5838
EW8
Percent Confluence
Time (hours)
0
24
30
48
60
72
90
96
120
144
150
168
180
192
210
240
288
336
DMSO
0.2 nM
0.5 nM
1 nM
2 nM

Fig. S1. Ewing sarcoma (EWS) cells are sensitive to genetic and pharmacological inhibition of NAMPT with OT-82, an efficacious, on-target NAMPT inhibitor. a Images of NeatStain (AstralDiagnostics, West Deptford, NJ) stained TC71 cells treated with negative control siRNA or 4 distinct siNAMPT sequences at 6 days post transfection. **b** Immunoblot analysis of NAMPT expression in EWS cell lines (TC32, and RDES) 72 hours after siRNA knockdown of NAMPT. **c** Cellular viability in EWS cell lines (TC32, and RDES), represented by percent confluence, at 6- and 4.5-days post transfection with NAMPT siRNA using siNAMPT sequence 6. **** denotes $p < 0.0001$**. d** Images of NeatStain stained TC32 and RDES cells treated with negative control siRNA, siNAMPT sequence 6, or siDeath control sequence at 6- and 4.5-days post transfection, respectively. **e** IncuCyte live-cell images of TC71, TC32, and RDES cells treated with DMSO or OT-82 at 2 nM at 72 hours. **f** IncuCyte live-cell analysis showing viability of EWS cell lines (CHLA-258, SK-N-MC, 5838, and EW8) as percent confluence after treatment with OT-82 at doses between 0.2 and 2 nM following overnight plating.

Fig. S2

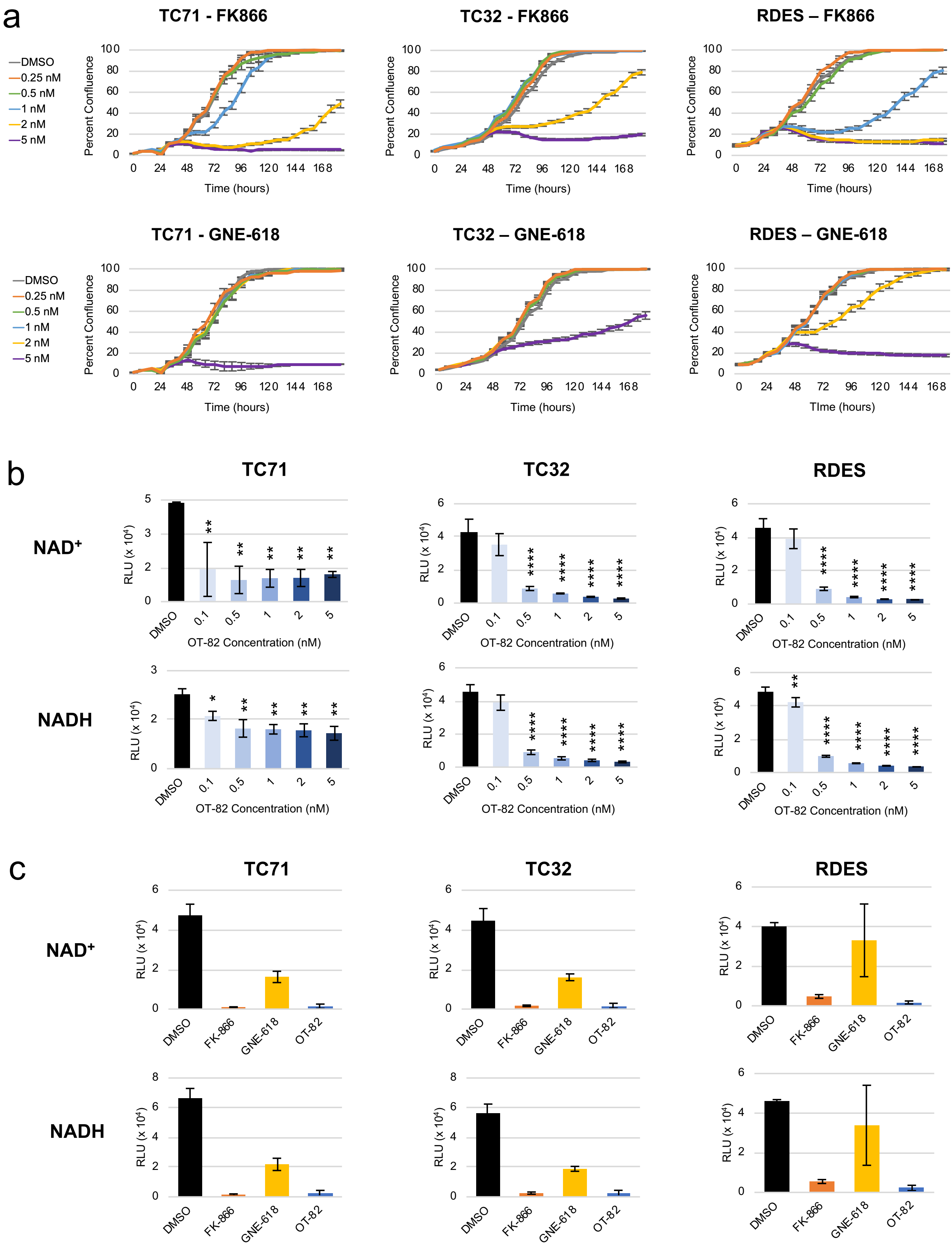
a
TC71 - FK866
TC32 - FK866
RDES – FK866
DMSO
0.25 nM
0.5 nM
1 nM
2 nM
5 nM
Percent Confluence
Time (hours)
TC71 - GNE-618
TC32 – GNE-618
RDES – GNE-618
TIme (hours)
b
TC71
TC32
RDES
NAD+
NADH
RLU (x 10⁴)
OT-82 Concentration (nM)
DMSO
0.1
0.5
1
2
5
c
TC71
TC32
RDES
NAD+
NADH
RLU (x 10⁴)
DMSO
FK-866
GNE-618
OT-82

Fig. S2. OT-82 is more potent than earlier generation NAMPT inhibitors FK866 and GNE-618. a IncuCyte live-cell analysis showing viability of EWS cell lines (TC71, TC32, and RDES) as percent confluence after treatment with DMSO, FK-866, or GNE-618 at concentrations between 0.25 and 5 nM following overnight plating. **b** NAD^+ and NADH concentrations in TC71, TC32, and RDES cells treated with DMSO or OT-82 at doses between 0.1 nM and 5 nM for 24 hours. * denotes $p < 0.05$, ** denotes $p < 0.01$, **** denotes $p < 0.0001$ for one-way ANOVA with Dunnett's multiple comparisons test. **c** NAD^+ and NADH concentrations in TC71, TC32, and RDES cells treated with DMSO, FK-866, GNE-618, or OT-82 at 0.5 nM for 72 hours.

Fig. S3

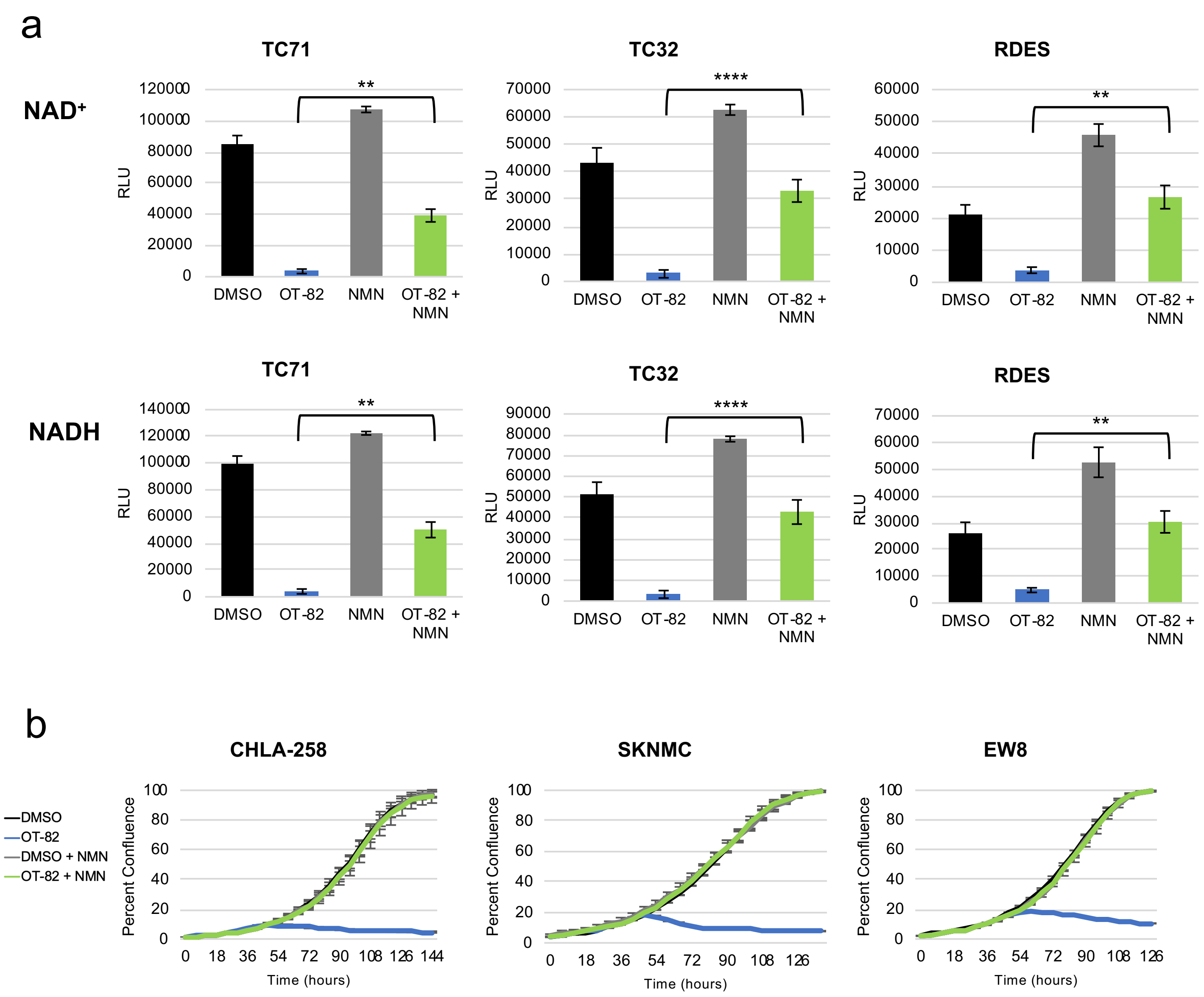

Fig. S3. NMN rescues the effects of OT-82 on NAD depletion and cellular viability. a NAD+ and NADH concentrations in TC71, TC32, and RDES cells treated with DMSO, NMN at 1 mM, OT-82 at 5 nM, or OT-82 plus NMN after 72 hours. ** denotes $p < 0.01$, **** denotes $p < 0.0001$ for comparison of OT-82 and OT-82 + NMN groups. **b** IncuCyte live-cell analysis showing viability of EWS cell lines (CHLA-258, SK-N-MC, and EW8) as percent confluence after treatment with DMSO, NMN at 1 mM, OT-82 at 5 nM, or OT-82 plus NMN, following overnight plating.

Fig. S4

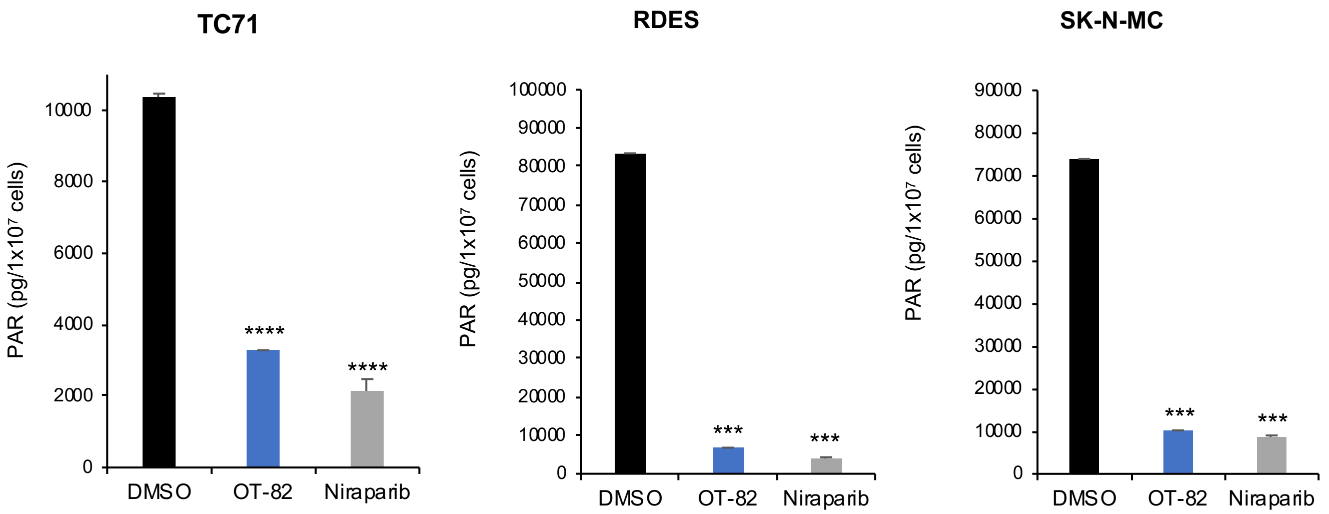

Fig. S4. OT-82 impairs PARP activity as effectively as a direct PARP inhibitor. PARylation of TC71, RDES, and SK-N-MC cells treated with DMSO, OT-82 at 5 nM for 24 hours, or the PARP inhibitor niraparib at 1 uM for 6 hours. *** denotes p <0.001, **** denotes p < 0.0001 for comparison of DMSO to either treatment group.

Fig. S5

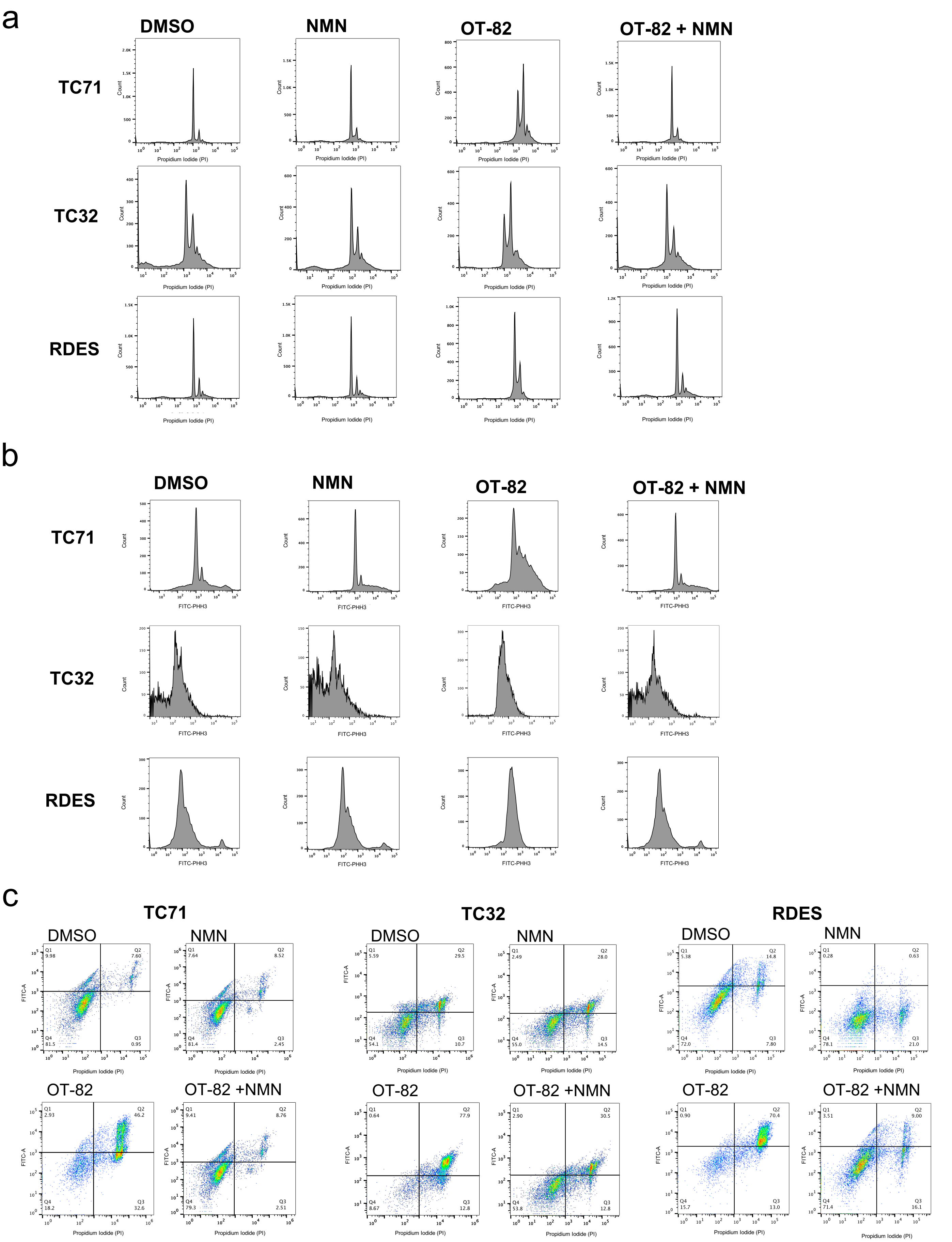
a
DMSO
NMN
OT-82
OT-82 + NMN
TC71
TC32
RDES
Count
Propidium Iodide (PI)
b
DMSO
NMN
OT-82
OT-82 + NMN
TC71
TC32
RDES
Count
FITC-PHH3
c
TC71
TC32
RDES
DMSO
NMN
OT-82
OT-82 +NMN
FITC-A
Propidium Iodide (PI)
Q1
Q2
Q3
Q4

Fig. S5. OT-82 induces mitotic arrest and cell death in EWS cells. a Representative histograms showing the changes in cell cycle fractions for TC71, TC32, and RDES cells treated with DMSO, NMN at 1 mM, OT-82 at 5 nM, or OT-82 plus NMN for 72 hours. **b** Representative histograms depicting event count of cells positively stained with both rabbit anti-phospho-Histone H3 (S10) and secondary anti-rabbit Alexa fluor 488 conjugated antibodies for TC71, TC32, and RDES cells treated with DMSO, NMN at 1 mM, OT-82 at 5 nM, or OT-82 plus NMN for 72 hours. Unstained cells represented by the large peak on the left and phospho-histone H3 represented by the smaller peak on the right. **c** Representative flow plots showing the changes in cellular fraction expressing annexin V and/or PI staining for TC71, TC32, and RDES cells treated with DMSO, NMN at 1 mM, OT-82 at 5 nM, or OT-82 plus NMN for 72 hours.

Fig. S6

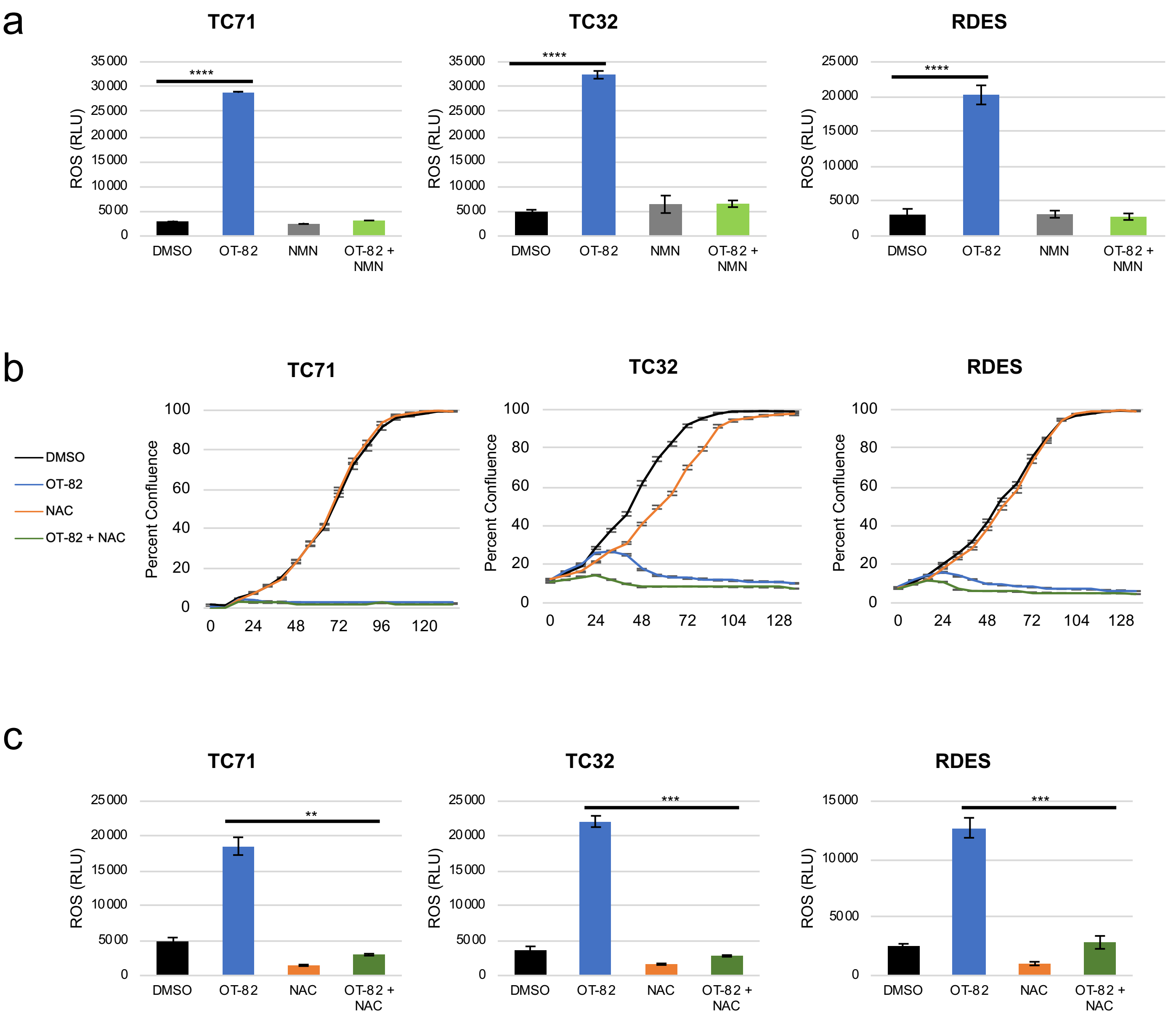

Fig. S6. ROS is a byproduct of OT-82 induced cell death in EWS cells. a ROS levels in TC71, TC32, and RDES cells treated with DMSO, OT-82 at 5 nM, NMN at 1 mM, or OT-82 plus NMN for 72 hours. **** denotes $p < 0.0001$ for comparison of DMSO and OT-82 groups. **b** IncuCyte live-cell analysis showing viability of EWS cell lines (TC71, TC32, and RDES) as percent confluence after treatment with DMSO, OT-82 at 5 nM, N-acetylcysteine (NAC) at 5 mM or OT-82 plus NAC following overnight plating. **c** ROS levels in TC71, TC32, and RDES cells treated with DMSO, OT-82 at 5 nM, NAC at 5 mM or OT-82 plus NAC for 72 hours. ** denotes $p <0.01$, *** denotes $p < 0.001$ for comparison of OT-82 and OT-82 + NAC groups.

Fig. S7

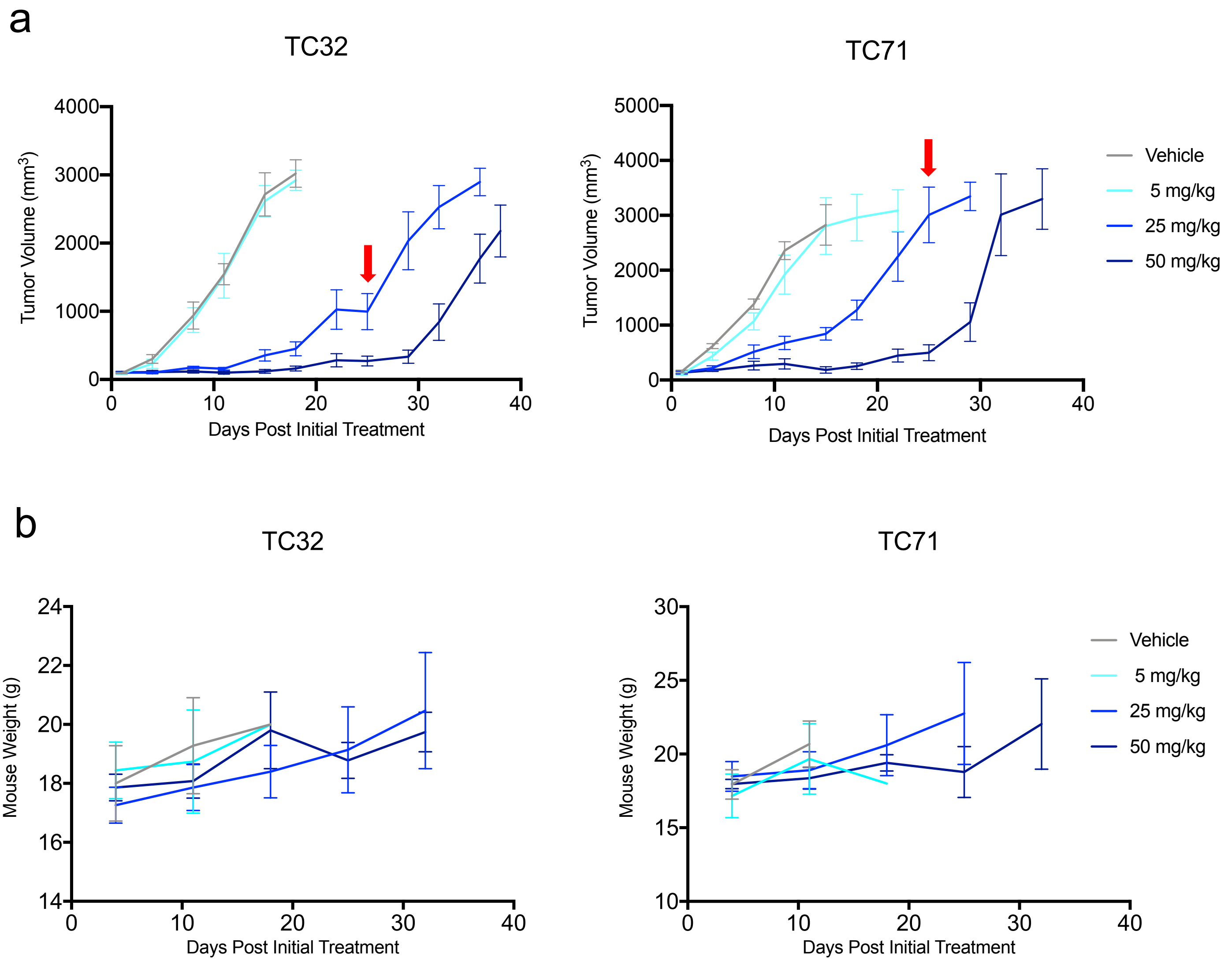

Fig. S7. OT-82 treatment does not cause weight loss in mice. a Tumor growth curves for TC32- and TC71-bearing xenografts (n=5/group) treated with vehicle or OT-82 at 5-, 25-, or 50-mg/kg. Red arrows represent final day of treatment. **b** Average weights of animals bearing EWS xenografts and treated with vehicle or OT-82 at 5-, 25- or 50-mg/kg.

Fig. S8

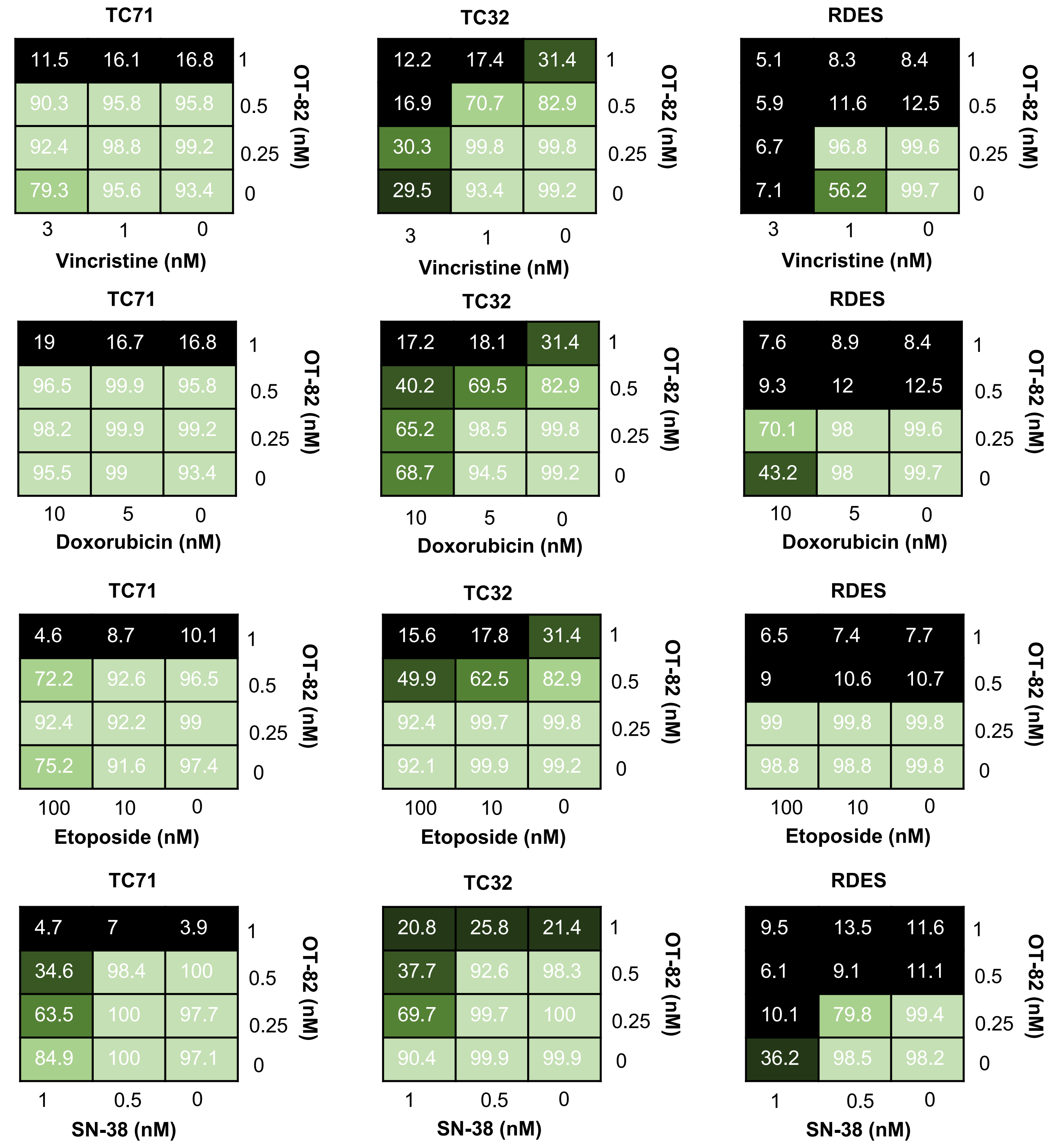

Fig. S8. Different chemotherapeutic agents differentially enhance the antiproliferative activity of OT-82 in EWS cell lines. Combination drug-treatment matrices showing percent confluence after treatment with vincristine (0-3 nM), doxorubicin (0-10 nM), etoposide (0-100 nM), and SN-38 (0-1 nM) alone and in combination with low-dose OT-82 (0-1 nM). Percent confluence (numeric values in each box) was measured using IncuCyte live-cell analysis at 96 hours of treatment, following overnight plating.

Fig. S9

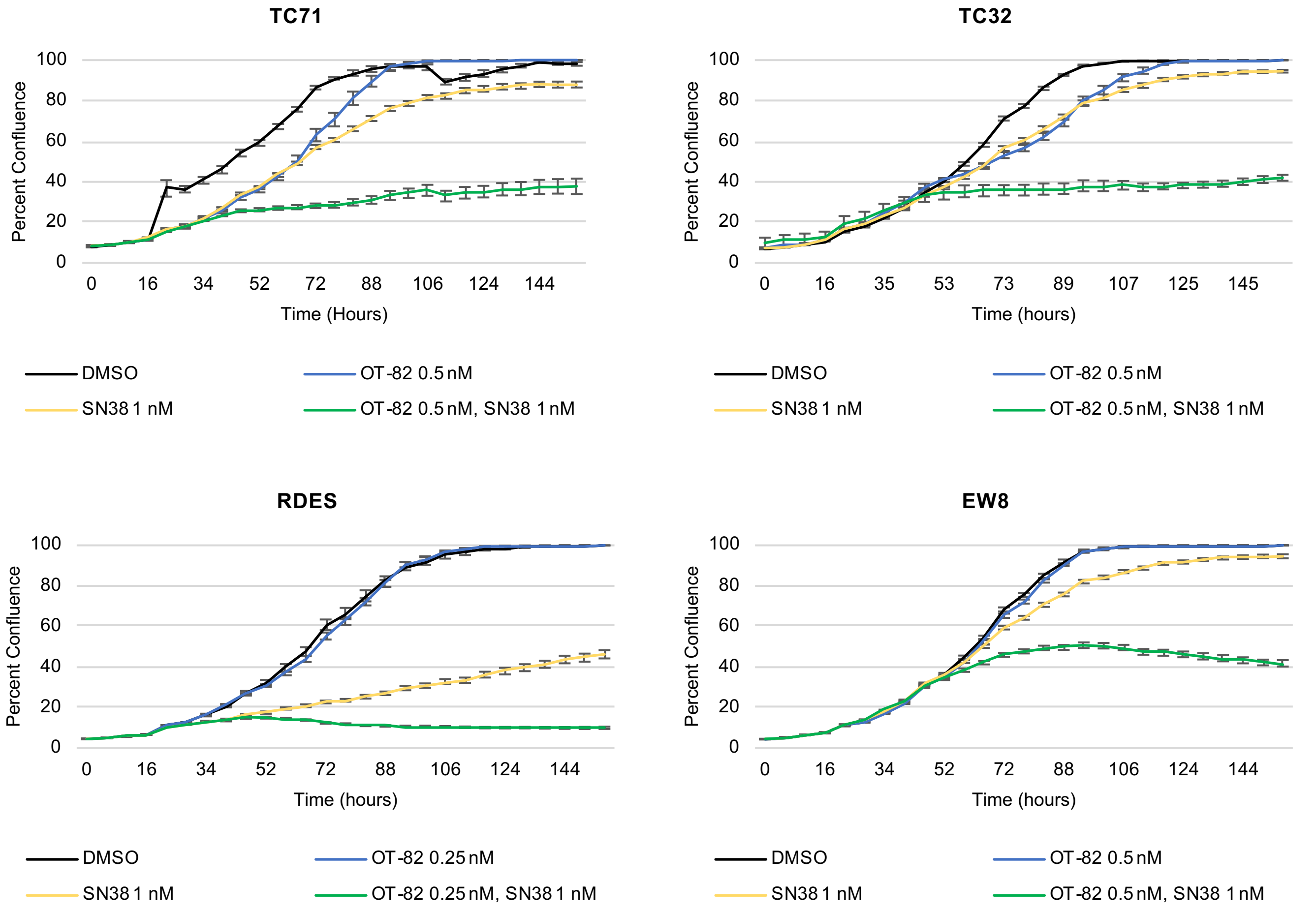

Fig. S9. Combination treatment with topoisomerase 1 inhibition improves single agent efficacy of OT-82 in EWS cell lines. IncuCyte live-cell analysis showing viability of EWS cell lines (TC71, TC32, RDES and EW8) as percent confluence after treatment with DMSO, low-dose OT-82, low-dose SN-38, or the combination, at indicated concentrations, following overnight plating.

Fig. S10

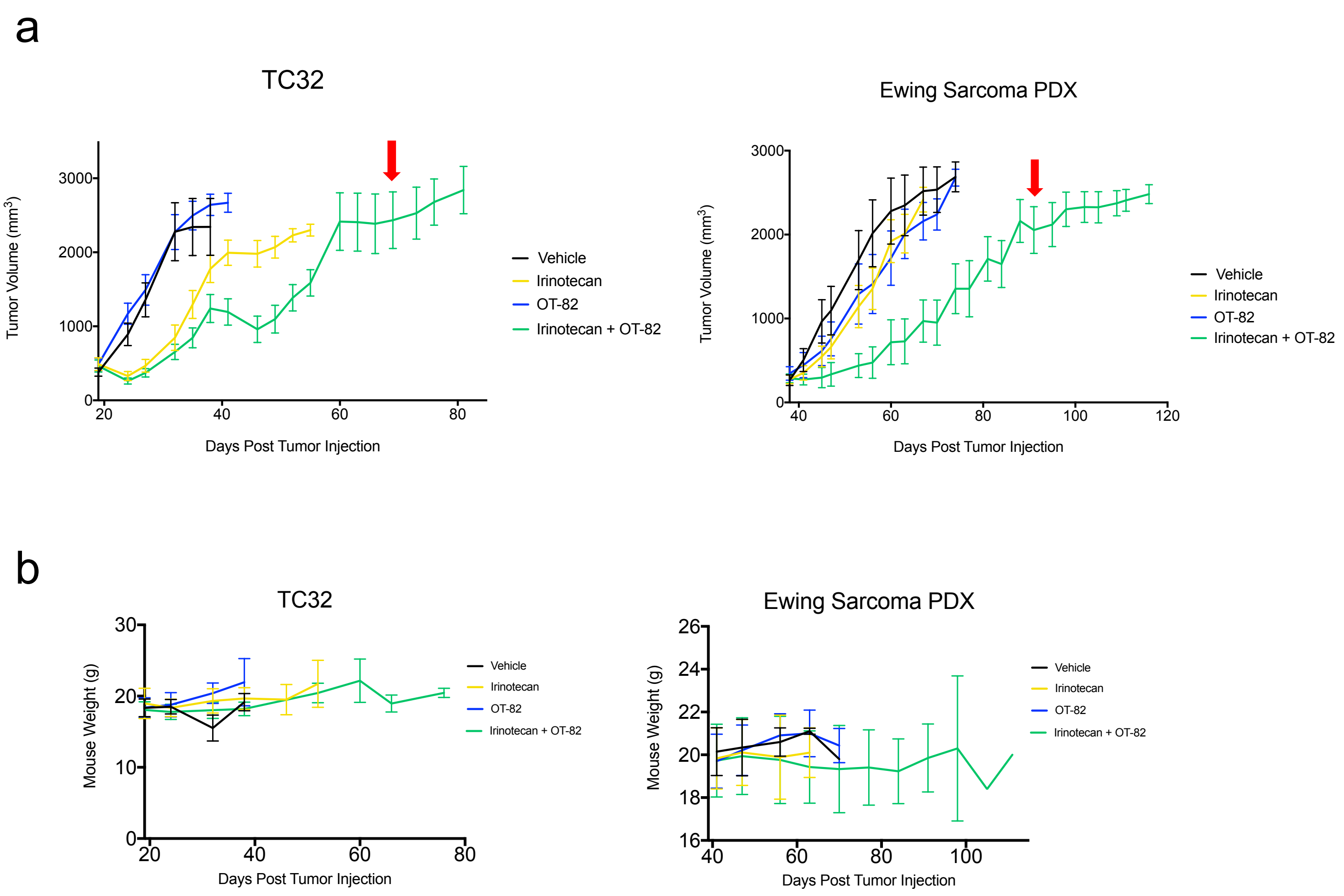

Fig. S10. Combination treatment with topoisomerase 1 inhibition improves single agent efficacy of low-dose OT-82 in EWS xenograft models. a Tumor growth curves for TC32- and PDX-bearing xenografts (n=12/group) treated with vehicle, low-dose OT-82 (15-mg/kg for TC32; 25-mg/kg for PDX), low-dose irinotecan (5 mg/kg daily on 2 of every 7 days for TC32; 1.25 mg/kg daily on 5 of every 7 days for PDX) or the combination. Red arrows represent final day of treatment. **b** Average weights of animals bearing TC32 or PDX xenografts and treated as described in a.

Fig. S11

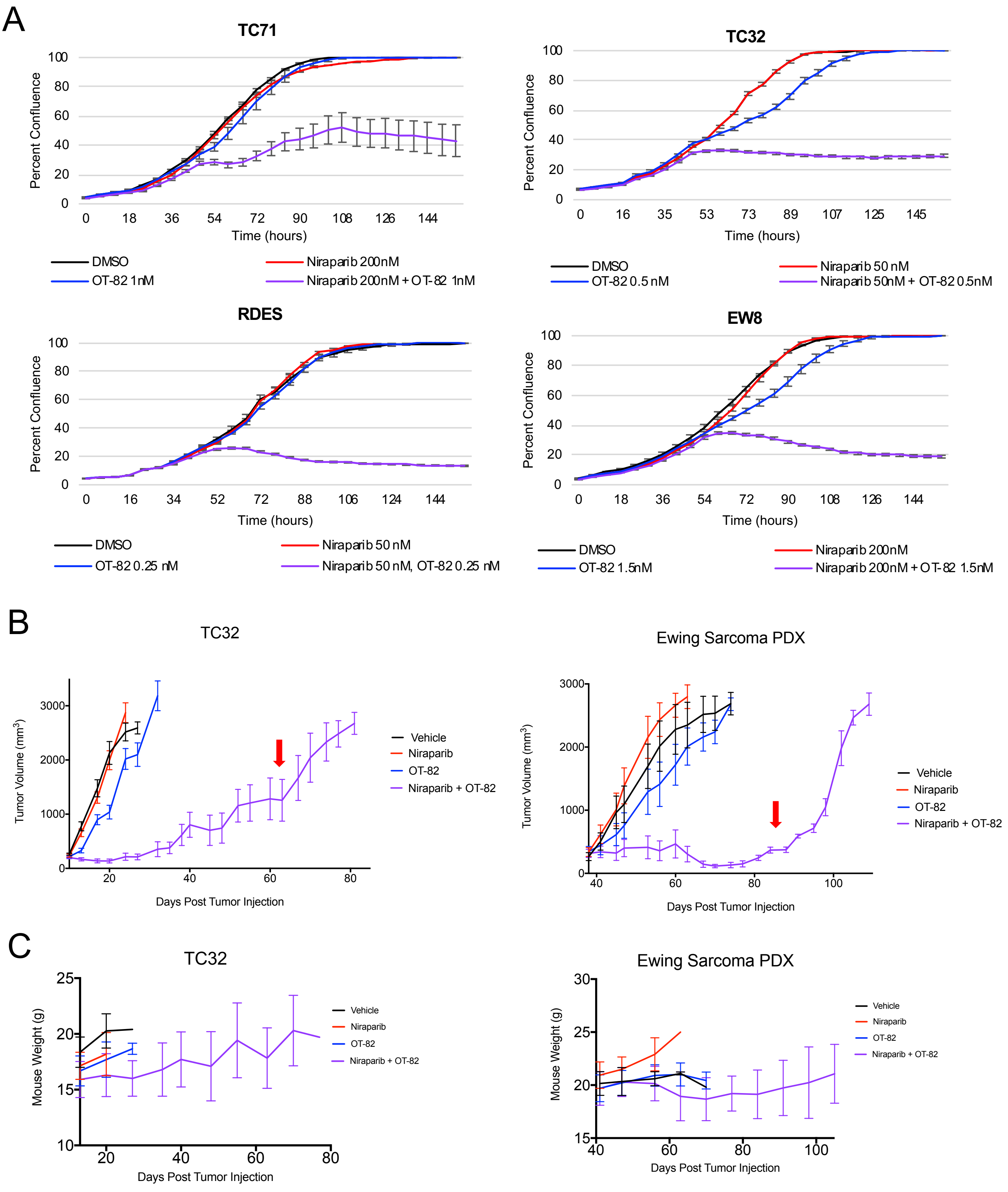
A
TC71
Percent Confluence
Time (hours)
DMSO
OT-82 1nM
Niraparib 200nM
Niraparib 200nM + OT-82 1nM
TC32
DMSO
OT-82 0.5 nM
Niraparib 50 nM
Niraparib 50nM + OT-82 0.5nM
RDES
DMSO
OT-82 0.25 nM
Niraparib 50 nM
Niraparib 50 nM, OT-82 0.25 nM
EW8
DMSO
OT-82 1.5nM
Niraparib 200nM
Niraparib 200nM + OT-82 1.5nM
B
TC32
Ewing Sarcoma PDX
Tumor Volume (mm^3)
Days Post Tumor Injection
Vehicle
Niraparib
OT-82
Niraparib + OT-82
C
TC32
Ewing Sarcoma PDX
Mouse Weight (g)
Days Post Tumor Injection
Vehicle
Niraparib
OT-82
Niraparib + OT-82

Fig. S11. Combination treatments with PARP inhibition improves single agent efficacy of low-dose OT-82 in EWS xenograft models. a IncuCyte live-cell analysis showing viability of EWS cell lines (TC71, TC32, RDES and EW8) as percent confluence after treatment with DMSO, OT-82, niraparib, or the combination, at indicated concentrations, following overnight plating. **b** Tumor growth curves for TC32- and PDX-bearing xenografts (n=12/group) treated with, low-dose OT-82 (15-mg/kg for TC32; 25-mg/kg for PDX), low-dose niraparib (50-mg/kg daily on 5 of every 7 days) or the combination. Red arrows represent final day of treatment. **c** Average weights of animals bearing TC32 or PDX xenografts and treated as described in b.
