# Supplementary material for: Inhibition of nicotinamide phosphoribosyltransferase (NAMPT) with OT-82 induces DNA damage, cell death, and suppression of tumor growth in preclinical models of Ewing sarcoma
Source: Oncogenesis. 2020 Sep 10;9(9):80. doi: 10.1038/s41389-020-00264-0 (PMC7481307; doi:10.1038/s41389-020-00264-0)
Supplement: Supplementary file 2 — Supplemental Methods [file 41389_2020_264_MOESM2_ESM.pdf]

Inhibition of nicotinamide phosphoribosyltransferase (NAMPT) with OT-82 induces DNA damage, cell death, and suppression of tumor growth in preclinical models of Ewing sarcoma

Anna E. Gibson, Choh Yeung, Sameer H. Issaq, Victor J. Collins, Michael Gouzoulis, Yiping Zhang, Jiuping Ji, Arnulfo Mendoza, Christine M. Heske

Supplemental Methods

Compounds

Stock solutions for *in vitro* experiments were prepared in DMSO, aliquoted, and stored at -20°C. For *in vivo* use, OT-82 powder was dissolved in a solution of 30% (2)-Hydroxypropyl-B-cyclodextrin (HPBCD) (MilliporeSigma, St. Louis, MO) with 40 mM HCl. Niraparib was dissolved in 20% HPBCD for *in vivo* use. Irinotecan was diluted to desired concentration with sterile saline prior to animal injection.

Immunoblotting

Cells were plated at 10^6/10-cm plate. Upon harvest, cells were washed with ice-cold PBS (ThermoFisher Scientific, Grand Island, NY), and lysed with cell lysis buffer (Cell Signaling Technology, Danvers, MA) with phosphatase/protease inhibitors (ThermoFisher). Protein lysates (30 µg/lane) were separated in 4%-12% SDS-PAGE (ThermoFisher) and transferred to nitrocellulose membranes (ThermoFisher). Membranes were blocked with 5% nonfat dry milk in TBS (KPL)-Tween 20 (MilliporeSigma; 25 mm Tris-HCl, pH 7.4; 0.13M of sodium chloride; 0.1% Tween 20).

PAR Immunoassay

Cells were plated at 2.5x10^6/10-cm plate overnight before treatment. OT-82 and DMSO were applied for 24 hours; niraparib treatments were applied for six hours, based on prior data indicating maximal inhibition at this timepoint (41). At harvest, cells were washed twice with ice-cold PBS (ThermoFisher), then lysed with cell lysis buffer (Cell Signaling Technology) with

phosphatase/protease inhibitors (ThermoFisher). For tissue-based experiments, 20 mg of frozen tumor was resuspended in 0.5 mL Cell Extraction Buffer (Invitrogen, Carlsbad, CA) supplemented with protease inhibitor (MilliporeSigma) and homogenized with a PRO200 homogenizer with a 5-mm probe (ProScientific, Oxford, CT) in an ice bath. Lysates were incubated on ice for 30 minutes prior to adding sodium dodecyl sulfate (Invitrogen) to a final concentration of 1%. Tubes were boiled for 5 minutes. Lysates were clarified by centrifugation at 12 000 × *g* for 5 minutes at 2-8°C. Protein levels were determined by BCA assay (ThermoFisher).

Cell Cycle Analysis

Cells were plated at 10^6/10-cm plate overnight before treatment. At 72 hours, cells were collected by scraping in the media and placed on ice. Plates were washed with 5 ml of cold PBS, and wash was pooled with collected cells and media. Cells were centrifuged at 500 x g for 5 minutes at 4°C, washed once with PBS, resuspended in cold PBS, and fixed with cold 70% ethanol/PBS overnight at -20°C. Cells were centrifuged at 500 x g for 10 minutes, washed with cold PBS, resuspended in PI/Triton X-100 staining solution (0.1% Triton X-100 (MilliporeSigma) in PBS, DNAase-free RNAse A (MilliporeSigma), PI (ThermoFisher)), then incubated at 37°C for 15 minutes before filtering through a Corning strainer tube.

Phospho-Histone H3 (Ser10) Staining

Cells were plated, collected, washed, spun, and fixed as described above. After overnight fixing, cells were centrifuged at 500 x g for 10 minutes, washed with PBS, resuspended in PBS/0.25% Triton X-100, and incubated on ice for 15 minutes. Cells were centrifuged at 500 x g for 5 minutes, washed with PBS, resuspended in 1:1 000 anti-H3 antibody (Cell Signaling Technology) in 1% BSA/PBS, and incubated overnight. 1% BSA/PBS was added to cells before spinning down. Cell pellet was resuspended in anti-rabbit Alexa 488 (ThermoFisher) at 1:250 in 1% BSA/PBS and incubated at room temperature for 30 minutes in the dark. PBS was added, and cells were spun down, then resuspended in PBS with RNAase A and incubated for 30 minutes at 37°C in the dark. PBS with 25 µg/ml propidium iodide was added before cells were strained and run on the flow cytometer.

Animal Studies

For cell line xenografts, two million cells were resuspended in cold HBSS and injected orthotopically into the left gastrocnemius muscle. For PDX experiments, PDX cells were thawed and put into Geltrex LDEV-free reduced growth factor basement membrane matrix (ThermoFisher), mixed at a 1:1 ratio with HBSS. 100 µL of this solution was injected orthotopically into the gastrocnemius muscle in the left hind leg. Mice were randomized after tumors were palpable. No blinding was performed.

Mice were treated with vehicle (HBPCD) or OT-82 by oral gavage daily

three consecutive days/week for four weeks in single-agent experiments (5 mice/group). For combination experiments, mice were treated with vehicle or OT-82 on the schedule described above for eight weeks (12 mice/group). Niraparib was given by oral gavage, five consecutive days/week for eight weeks. Irinotecan was given by intraperitoneal (IP) injection twice/week for eight weeks for TC32 xenografts and for five consecutive days/week for eight weeks for PDX.
